# The contribution of the tendon electrode to M‐wave characteristics in the biceps brachii, vastus lateralis and tibialis anterior

**DOI:** 10.1113/EP091472

**Published:** 2023-11-21

**Authors:** Javier Rodriguez‐Falces, Saioa Etxaleku, Gabriel S. Trajano, Igor Setuain

**Affiliations:** ^1^ Department of Electrical and Electronical Engineering Public University of Navarra Pamplona Spain; ^2^ Clinical Research Department TDN, Orthopedic Surgery and Advanced Rehabilitation Center Mutilva Spain; ^3^ Faculty of Health, School of Exercise and Nutrition Sciences Queensland University of Technology (QUT) Brisbane Queensland Australia; ^4^ Department of Health Sciences Public University of Navarre Pamplona Spain

**Keywords:** active electrode, belly–tendon configuration, compound muscle action potential, far‐field potentials, M wave, reference electrode, tendon potential, volume conduction

## Abstract

In some compound muscle action potentials (M waves) recorded using the belly–tendon configuration, the tendon electrode makes a noticeable contribution to the M wave. However, this finding has only been demonstrated in some hand and foot muscles. Here, we assessed the contribution of the tendon potential to the amplitude of the vastus lateralis, biceps brachii and tibialis anterior M waves, and we also examined the role of this tendon potential in the shoulder‐like feature appearing in most M waves. M waves were recorded separately at the belly and tendon locations of the vastus lateralis, biceps brachii and tibialis anterior from 38 participants by placing the reference electrode at a distant (contralateral) site. The amplitude of the M waves and the latency of their peaks and shoulders were measured. In the vastus lateralis, the tendon potential was markedly smaller in amplitude (∼75%) compared to the belly M wave (*P* = 0.001), whereas for the biceps brachii and tibialis anterior, the tendon and belly potentials had comparable amplitudes. In the vastus lateralis, the tendon potential showed a small positive peak coinciding in latency with the shoulder of the belly–tendon M wave, whilst in the biceps brachii and tibialis anterior, the tendon potential showed a clear negative peak which coincided in latency with the shoulder. The tendon potential makes a significant contribution to the belly–tendon M waves of the biceps brachii and tibialis anterior muscles, but little contribution to the vastus lateralis M waves. The shoulder observed in the belly–tendon M wave of the vastus lateralis is caused by the belly potential, the shoulder in the biceps brachii M wave is generated by the tendon potential, whereas the shoulder in the tibialis anterior M wave is caused by both the tendon and belly potentials.

## Abstract (highlights)

- **What is the central question of this study?**
  Does a tendon electrode make a noticeable contribution to the belly–tendon M wave in the vastus lateralis, biceps brachii and tibialis anterior muscles?
- **What is the main finding and its importance?**
  Because the patellar tendon potential is small in amplitude, it hardly influences the amplitude and shape of the belly–tendon M wave of the vastus lateralis. However, for the biceps brachii and tibialis anterior muscles, the potentials at the tendon sites show a large amplitude, and thus have a great impact on the corresponding belly–tendon M waves.

## 1 INTRODUCTION

**FIGURE 1:**
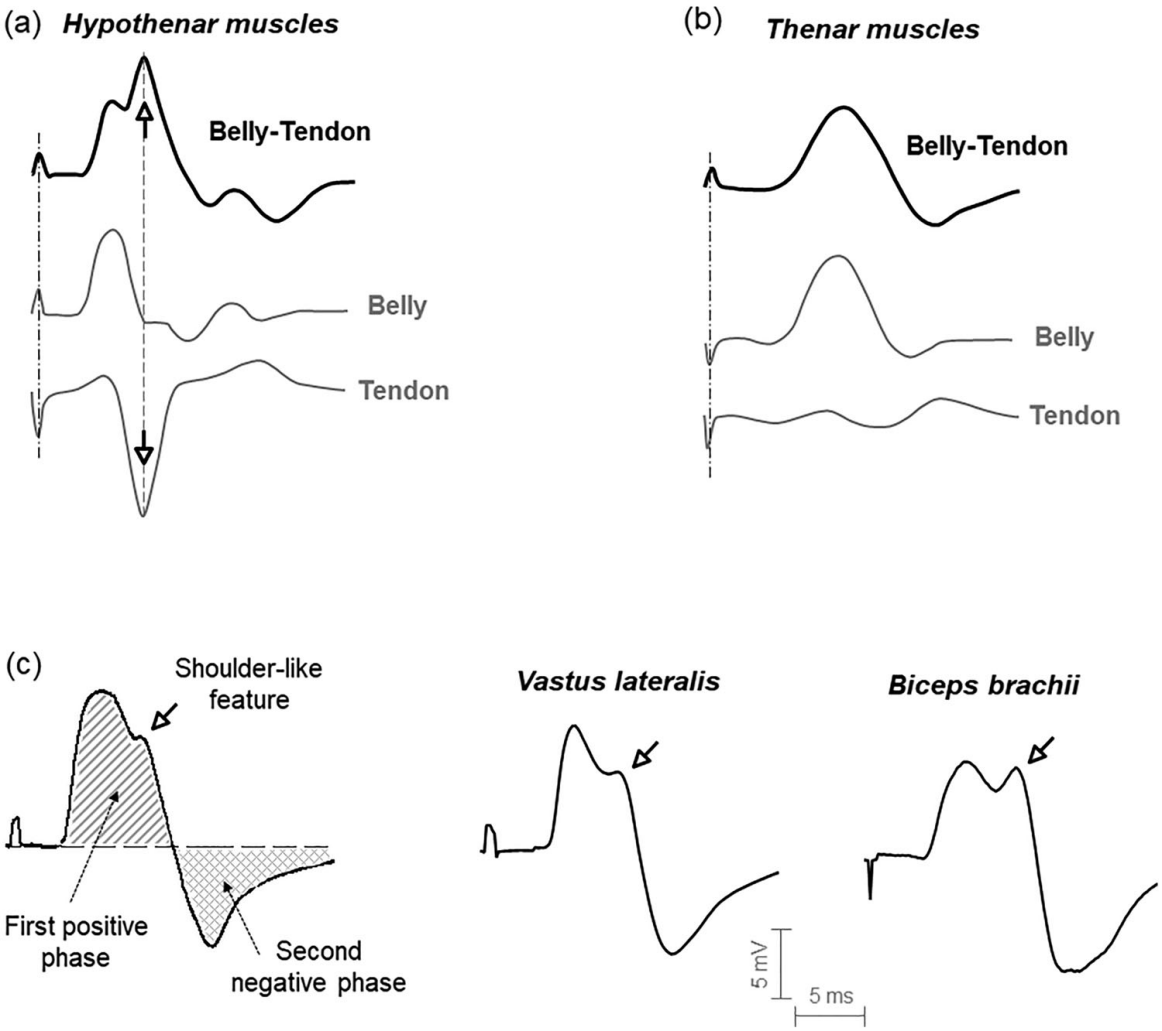
(a, b) Representative examples of belly–tendon M waves (upper black trace in each plot) together with their components at the belly and tendon sites (lower grey traces) recorded in the hypothenar (a) and thenar (b) muscles. (c) Representative examples of M waves recorded in the vastus lateralis and biceps brachii using the belly–tendon montage. Note the shoulder‐like feature appearing in the descending portion of the M wave first phase (white arrows).

When compound muscle action potentials (M waves) are recorded using the belly–tendon configuration, the belly electrode is placed over the motor point on the muscle belly, whereas the reference electrode is usually located at the muscle's distal tendon. For a long time, it was assumed that the tendon location is electrically inactive, and thus all the electrical activity is theoretically picked up by the belly electrode (Hammer, 1982; Weber, 1988). However, this assumption was proved incorrect by Kincaid et al. (1993), who, by placing the reference electrode at a distant (contralateral) site, were able to record separately the contributions of the belly and tendon electrodes (see examples of Figure 1). The electrical activity recorded by the tendon electrode is mainly far‐field potentials generated by dipole sources created after stimulation of the peripheral nerve (Stegeman et al., 1997). This is possible because far‐field (stationary) components have the same amplitude irrespective of the distance to the source. In contrast, the belly electrode picks up near‐field potentials emerging from moving quadrupoles (i.e., action potentials propagating along muscle fibres) as well as far‐field potentials from dipole sources. Despite this theoretical knowledge, the shape and amplitude characteristics of the tendon potential vary greatly from muscle to muscle, and there is not yet a satisfactory explanation for such differences.

The first point that remains unclear is why the potential at the tendon site is of high amplitude (compared to the belly potential) after stimulating some peripheral nerves (as shown in Figure 1a), whereas this tendon site shows only a low amplitude potential following stimulation of other nerves (as shown in Figure 1b). Specifically, a large tendon potential was registered for the hypothenar muscles (ulnar nerve stimulation) and abductor hallucis (tibial nerve stimulation), whilst this tendon potential was significantly smaller than the belly potential for the thenar muscles (median nerve) and extensor digitorum brevis (peroneal nerve) (Brashear & Kincaid, 1996; Kincaid et al., 1993; Nandedkar & Barkhaus, 2007). Importantly, the tendon potential and its contribution to the belly–tendon M wave has only been studied in a few muscles of the hand and foot (Kincaid et al., 1993; Nandedkar & Barkhaus, 2007; Van Dijk et al., 1999), and it would be highly valuable to examine the tendon contribution for larger muscles more commonly used in sport science disciplines. Thus, in the present experiments, we extend this line of investigation to the vastus lateralis, biceps brachii and tibialis anterior.

The second point to address is the electrical origin of the shoulder‐like feature (or second peak) observed in the descending portion of the first phase of some belly–tendon M waves (for examples, see Figure 1c). The presence of this ‘shoulder’ was first reported by Kincaid et al. (1993) in the hypothenar muscles’ M waves, and attracted the attention of subsequent authors (Nandedkar & Barkhaus, 2007; Van Dijk et al., 1999). More recently, our group has demonstrated that the shoulder is also present in most belly–tendon M waves recorded in the vastus lateralis (Rodriguez‐Falces & Place, 2018) and biceps brachhi (Rodriguez‐Falces et al., 2021). Interestingly, Kincaid and coworkers argued that the shoulder was present only in those muscles in which the tendon potential makes a significant contribution to the belly–tendon M wave and, what is more, they theorized that the shoulder was indeed generated by the tendon potential. To support this hypothesis, the authors offered the following example: the presence of the shoulder in the hypothenar M waves was due to the large tendon potential recorded following ulnar nerve stimulation (Figure 1a) and, conversely, the lack of shoulder in the thenar M waves was consistent with the low‐amplitude tendon potential generated after median nerve stimulation (Figure 1b) (Kincaid et al., 1993). However, this hypothesis has so far been tested only in hand and foot muscles, and it may be that in larger muscles the presence of the shoulder in the belly–tendon M waves is not related to the contribution of the tendon potential.

The necessity of examining the belly and tendon potentials at locations different from the foot and hand is also justified by the unique peculiarities of the electrical field in these regions. Indeed, in the hand and foot, there are many muscles tightly packed in a small space, and thus the belly electrode may not only record the near‐field activity from the muscle directly underneath, but also from neighbouring muscles through volume conduction (Van Dijk et al., 1999). In addition, in the hand and foot, the tendon electrode is normally placed in close proximity to various muscles, and thus it is likely that this electrode picks up near‐field electrical activity from these muscles. Apart from near‐field potentials, it has been shown that several far‐field (stationary) components appear in the hand following stimulation of the median and radial nerves (Kimura et al., 1984; Spaans, 1984). The dipole sources responsible for this far‐field activity are likely created as the moving source passes through a transition of volume conductor geometry (Kimura et al., 1984).

The objectives of the present study were: (1) to examine and quantify the actual contribution of the tendon electrode to the belly–tendon M waves of the vastus lateralis, biceps brachii and tibialis anterior, and (2) to test the hypothesis that, in the belly–tendon M waves, the shoulder‐like feature is due to the contribution of the potential recorded at the tendon site. These objectives were addressed by placing the reference electrode at the contralateral limb (i.e., at a distant site), which allowed separate recording of the potentials at the belly and tendon sites. This study was designed to gain insight into the formation of the belly–tendon M waves in three muscles commonly tested in sport science and rehabilitation research, as well as to provide recommendations on which electrode configuration, belly–tendon or bipolar, is best suited in each muscle.

## 2 METHODS

### 2.1 Ethical approval

Ethical approval was granted by the Ethical Committee of the Public University of Navarra, Spain (PI‐010/21) and the study conformed to the *Declaration of Helsinki*, except for registration in a database. A detailed summary of the methods and study design was given verbally to each participant before providing written assent. All participants provided written informed consent prior to participation.

### 2.2 Experimental set‐up

Thirty‐eight male participants aged between 23 and 33 years (mean ± SD: 25.3 ± 2.4 years) volunteered to participate in this study. Their average height and body mass were 178 ± 4 cm and 71 ± 5 kg, respectively. None of the subjects declared any current/recent musculoskeletal injuries or neuromuscular disorders.

Experiments consisted of evoking supra‐maximal M waves while the muscle was at rest in three different muscles using peripheral nerve stimulation. During the recordings on the vastus lateralis, participants were seated comfortably on a custom‐built chair with a trunk–thigh angle of 100°, a knee angle of 90°, while the ankle was securely strapped to a custom‐made mould. During the recordings on the biceps brachii, participants were seated with the shoulder 90° abducted, the elbow flexed at 120°, the forearm vertical and supinated, while the hand was holding an adjustable handle. During the recordings on the tibialis anterior, participants were seated on an adjustable chair in a slightly reclined position with the right foot strapped to a footplate. The plate was inclined at an angle of 45° relative to the floor and the seat was adjusted so that ankle and knee joint angles were at 90° and 120°, respectively.

### 2.3 Localization of the innervation zone

In each of the studied muscles, the position of the innervation zone was determined using a dry linear array of 16 electrodes (5×1 mm, 5 mm inter‐electrode distance), which was connected to a multichannel amplifier (OT Bioelettronica, Torino, Italy; bandwidth 10−500 Hz). The sEMG signals were registered in bipolar mode as the participant performed gentle isometric contractions. The main innervation zone was identified by observing the two bipolar channels showing phase reversal (Masuda et al., 1985).

### 2.4 Electromyographic recordings

**FIGURE 2:**
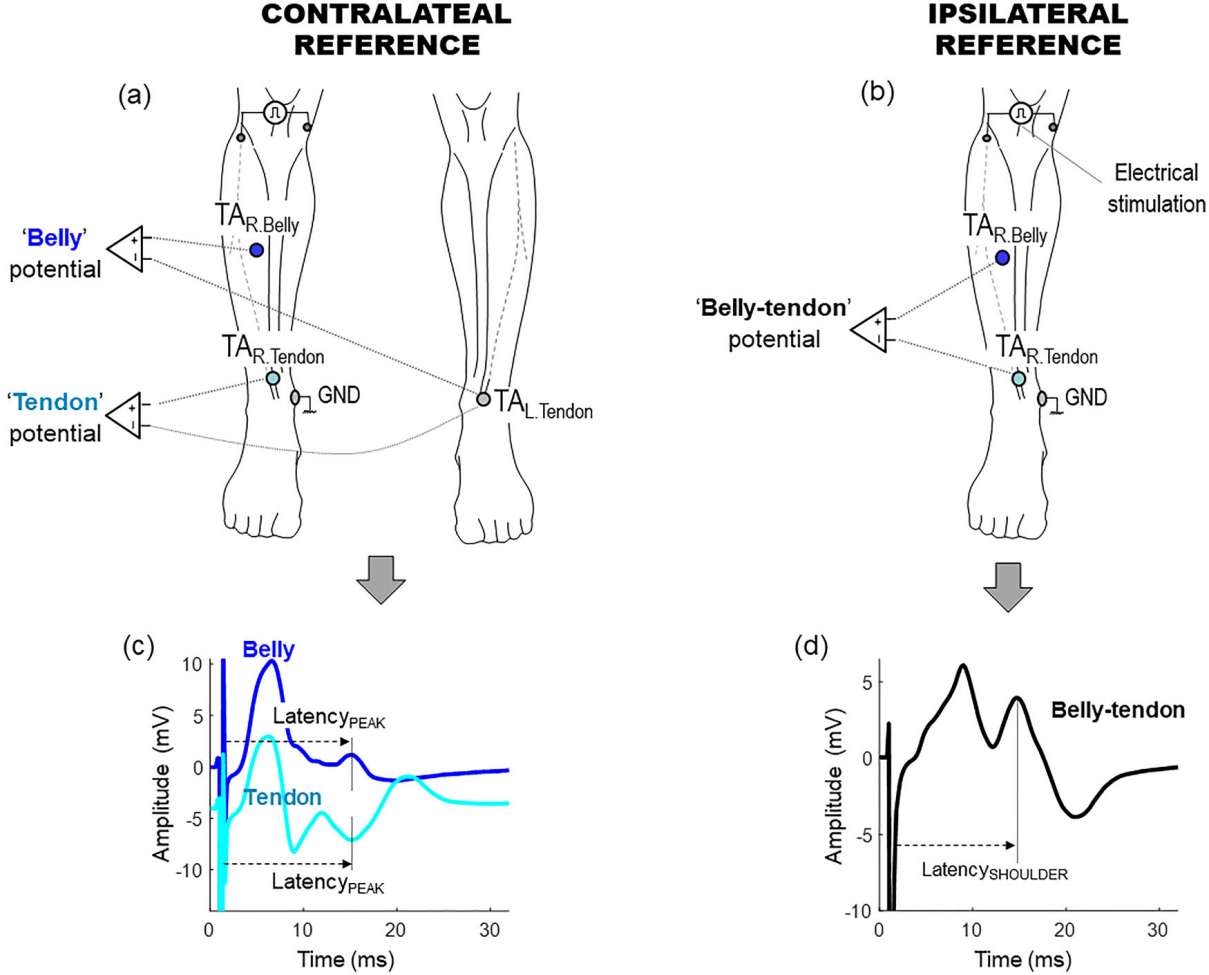
(a) Experimental arrangements adopted to record the potentials at the belly and tendon sites of the right tibialis anterior muscle, using the contralateral (left) distal tendon (TA_L.Tendon_) as reference site. (b) Experimental settings adopted to record ‘classical’ belly–tendon M waves in the right tibialis anterior, using the ipsilateral (right) tendon (TA_R.Tendon_) as reference site. (c) Representative examples of the belly and tendon potentials, together with the definition of the latency of the peak nearest to the latency of the shoulder of the belly–tendon M wave. (d) Representative example of the belly–tendon potential, together with the definition of the latency of it shoulder.

Surface electrodes were positioned at the belly and tendon sites of the vastus lateralis, biceps brachii and tibialis anterior of the right limb (e.g., see Figure 2). The ‘belly’ electrode was placed over the innervation zone of each muscle, as determined with the linear array described above. The tendon electrode corresponding to the vastus lateralis was placed over the patellar tendon, 3 cm below the kneecap (and the ground electrode over the kneecap). The tendon electrode of the biceps brachii was located on the antecubital fossa (and the ground electrode on the lateral epicondyle). As for the tibialis anterior, the tendon electrode was positioned on the distal tendon of this muscle (and the ground electrode over the medial malleolus).

To separately record the potentials at the belly and tendon sites, each site (electrode) on the right limb was referenced to an electrode placed on the contralateral (left) limb, as shown in Figure 2a, so that this contralateral electrode mirrored that of the ipsilateral limb. For example, in the left tibialis anterior, the contralateral reference electrode was placed over the distal tendon (TA_L.Tendon_). Hence, the belly potential of the right tibialis anterior was recorded as TA_R.Belly_ − TA_L.Tendon_, and the tendon potential were recorded as TA_R.Tendon_ − TA_L.Tendon_. It is assumed that any electrical activity at the contralateral reference site is minimal compared to that at the ipsilateral limb (Kincaid et al., 1993). The advantage of this contralateral reference system is that it allows recording of the whole electrical activity arising after stimulating a peripheral nerve, including the near‐ and far‐field components (Kincaid et al., 1993).

To record the M waves with the classical belly–tendon configuration, the belly electrode was referenced to an electrode placed over the tendon on the same (ipsilateral) limb, as shown in Figure 2b. For example, in the tibialis anterior, the belly–tendon M wave was recorded as the difference of activity between the belly and tendon sites on the same (right) leg, that is, TA_R.Belly_ − TA_R.Tendon_.

All surface EMG potentials were recorded using self‐adhesive surface Ag/AgCl electrodes (Kendall Meditrace 100, Tyco, Quebec, Canada) with circular shapes (recording diameter of 10 mm). Prior to electrode placement, the skin was adequately prepared (shaving, light abrasion with sandpaper and cleansing with rubbing alcohol) to reduce the skin–electrode impedance. Surface EMG signals were amplified (bandwidth, 10–1000 Hz) and digitized (sampling frequency, 5 kHz) using an analog‐to‐digital conversion system (MP150; Biopac Systems, Goleta, CA, USA).

### 2.5 Stimulation procedure

All peripheral nerves were stimulated using single rectangular pulse stimuli delivered by a high‐voltage (400 V maximum) constant‐current stimulator (DS7AH; Digitimer, Welwyn Garden City, UK). For stimulation of the femoral nerve, the cathode (5 cm diameter, Dermatrode, American Imex, Irvine, CA, USA) was placed at the femoral triangle, 3–5 cm below the inguinal ligament, and the anode (5 × 10 cm, Compex, Ecublens, Switzerland) was located over the gluteal fold, opposite the cathode (Rodriguez‐Falces & Place, 2017). For stimulation of the brachial plexus, the cathode (1 cm diameter, Kendall Meditrace 100) was positioned on the supraclavicular fossa, and the anode (3.5 × 4.5 cm, Compex) was located on the acromion (Lévénez et al., 2008). For stimulation of the deep peroneal nerve, the cathode (1 cm diameter, Kendall Meditrace 100) was placed over the deep peroneal nerve, close to the neck of the fibula, and the anode (3.5 × 4.5 cm, Compex) was fastened on the opposite side of the leg in the popliteal fossa (Place et al., 2009).

The maximal stimulus intensity was determined by gradually increasing the current intensity up to a level at which the M‐wave amplitude reached a plateau. This level of intensity was then further increased by 20% to ensure that the stimulation remained supramaximal throughout the experimental session (Neyroud et al., 2012). The pulse width was set at 1 ms for the femoral nerve (Rodriguez‐Falces & Place, 2017), and at 0.2 ms for the braxial plexus (Smith et al., 2007) and peroneal nerve (Place et al., 2009).

### 2.6 Convention for the polarity of the M‐wave phases

The M waves were plotted with negative voltages upward. Thus, in the following, the terms ‘positive’ and ‘negative’ were used with reference to the direction of the plot (as shown in Figure 1c), rather than the true electrical polarity.

### 2.7 Data analysis

Data were recorded with a commercially available software (AcqKnowledge, Biopac Systems), and subsequently data were exported to MATLAB (version R2012b; The MathWorks, Natick, MA, USA) for quantitative analysis using customized scripts.

The first objective of the study was to quantify the actual contribution of the tendon electrode to the belly–tendon M waves. To do so, the peak‐to‐peak amplitude (Ampli_PP_) of the belly, tendon and belly–tendon M waves was computed as the difference between the maximum positive and minimum negative peaks of the potential.

The second objective of the study was to ascertain whether the shoulder (or second peak) in the belly–tendon M waves was caused by the occurrence of a peak in either the tendon potential or the belly potential. To do this, for the belly–tendon M waves, we calculated the latency of the shoulder (Latency_SHOULDER_) as the time elapsed between the stimulus artifact and the occurrence of such shoulder, as shown in Figure 2d. For the belly and tendon potentials, we calculated the latency of the peak (Latency_PEAK_) as the time elapsed between the stimulus artifact and the occurrence of the peak nearest to the latency of the shoulder of the belly–tendon M wave, as shown Figure 2c.

### 2.8 Statistics

The Kolmogorov–Smirnov test was used to confirm that each of the M‐wave parameters analysed in the current study was normally distributed. To examine differences between the tendon, belly and belly–tendon M waves, a two‐way repeated‐measures ANOVA (electrode site (tendon, belly and belly–tendon) × muscle (vastus lateralis, biceps brachii and tibialis anterior)) was performed on Ampli_PP_, Latency_SHOULDER_ and Latency_PEAK_. For significant results, the Bonferroni adjusted pairwise test was used for post‐hoc testing. Significance was set at *P* < 0.05. Data are presented as means ± SD in the text, tables and figures.

## 3 RESULTS

### 3.1 Amplitude and shape characteristics of the belly, tendon and belly–tendon M waves

**FIGURE 3:**
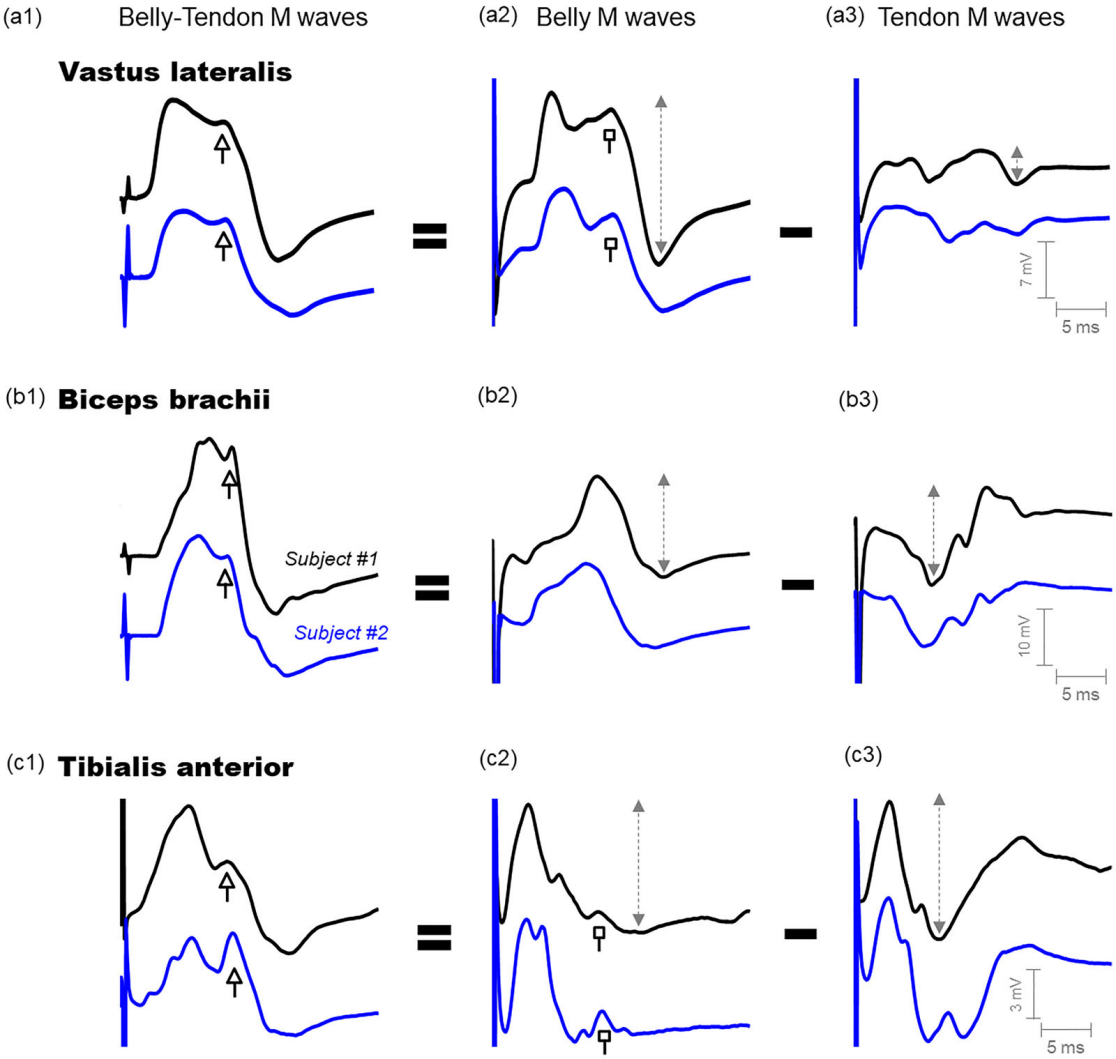
Representative examples of two belly–tendon M waves (first column), accompanied by their belly (second column) and tendon (third column) components, recorded from the vastus lateralis, biceps brachii and tibialis anterior of two participants. In each muscle, the belly–tendon M wave would be obtained as the belly potential minus the tendon potential (shown with the equals and minus symbols). The white arrows indicate the presence of the shoulder (or second peak) in the belly–tendon M waves. The white squares indicate the presence of shoulders or peaks in the belly potential.

Figure 3 shows representative examples of the belly–tendon M waves (first column), together with their corresponding belly (second column) and tendon (third column) components, recorded from the vastus lateralis, biceps brachii and tibialis anterior of two participants. It can be seen that all belly–tendon potentials showed a distinct shoulder‐like feature (or second peak) in the declining first phase (white arrows in Figures 3a, 1, b1 and c1). This shoulder was present in most of the vastus lateralis (96%), biceps brachii (93%) and tibialis anterior (91%) M waves analysed. Note that the belly potentials of the vastus lateralis also had clear shoulders (white squares in Figure 3a, 2), and those of the tibialis anterior exhibited a positive second peak (white squares in Figure 3c, 2).

**FIGURE 4:**
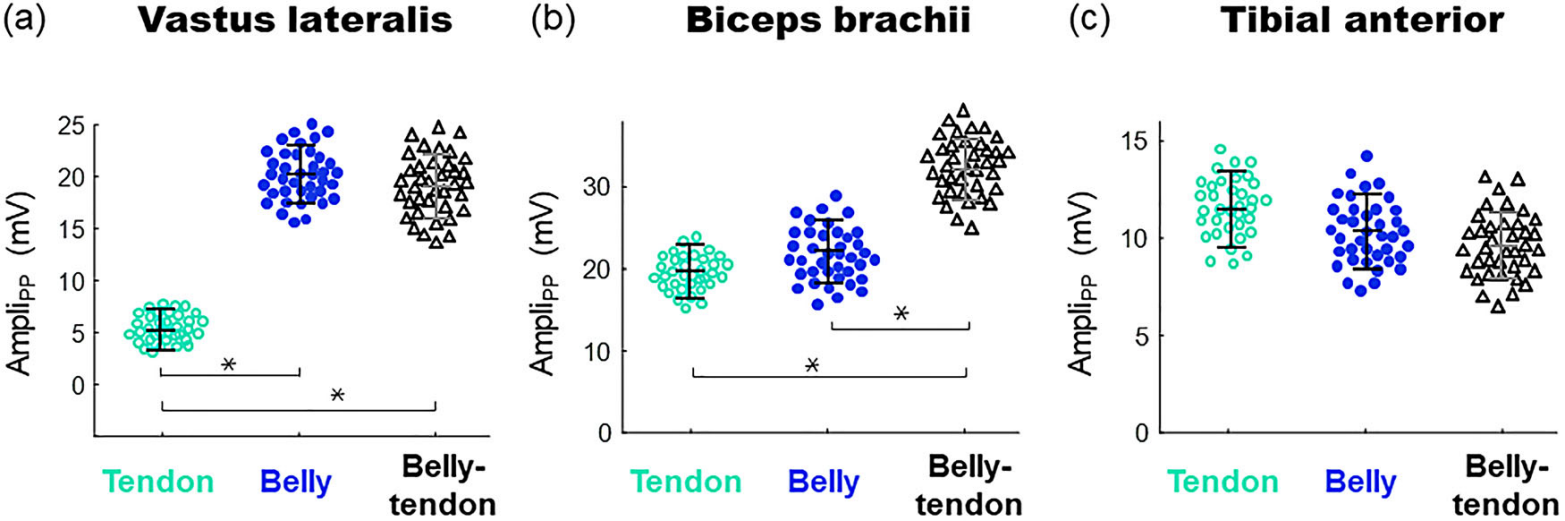
Mean and SD values of peak‐to‐peak amplitudes (Ampli_PP_) corresponding to the tendon, belly and belly–tendon M waves recorded in the vastus lateralis (a), biceps brachii (b), and tibialis anterior (c). *Significant difference in amplitude.

The amplitudes of the tendon, belly and belly–tendon M waves, averaged for the whole study group, are shown in Figure 4. For the vastus lateralis, the tendon potential was markedly smaller in amplitude (∼75%, *P* = 0.001, Table 1) compared to the belly and belly–tendon M waves, whereas the belly and belly–tendon potentials had similar amplitudes (see the first row of Figures 3 and 4a). For the biceps brachii, however, the tendon and belly potentials had comparable amplitudes, and these potentials were significantly smaller (∼40%, *P* = 0.003, Table 1) than the belly–tendon potentials (second row of Figures 3 and 4b). For the tibialis anterior, the tendon and belly potentials were comparable in magnitude, and these potentials had slightly greater amplitude than the belly–tendon potential (third row of Figures 3 and 4c). A significant interaction was found between muscle and electrode site (*P* = 0.02).

**TABLE 1:** Peak‐to‐peak amplitudes of the belly and tendon M waves normalized to the amplitude of the belly–tendon M waves amplitude and expressed as percentages.

	Relative amplitude (%)	
Muscle Muscles	Belly	Tendon
Vastus lateralis	106.4 ± 8.7	25.7 ± 6.8*
Biceps brachii	66.2 ± 8.3	58.6 ± 7.4
Tibialis anterior	110.9 ± 9.1	122.2 ± 9.3

*Note*: Values shown are means ± standard deviation, *n* = 38. *Significant difference in amplitude between the belly and tendon M waves.

As for the shape characteristics, the belly M waves of the three muscles all showed a biphasic waveform, namely, an initial positive phase followed by a negative phase (Figure 3a, 2, b2 and c2). In contrast, the shape of the tendon potentials varied from muscle to muscle. The quadriceps tendon potential showed a triphasic or quadriphasic waveform (negative–positive–negative–positive, Figure 3a3), the biceps brachii tendon potential had a biphasic shape (negative–positive, Figure 3b3), and the tibialis anterior tendon potential exhibited a triphasic shape (positive–negative–positive, Figure 3c3).

### 3.2 Contribution of the tendon and belly potentials to the shoulder of the belly–tendon M wave

**FIGURE 5:**
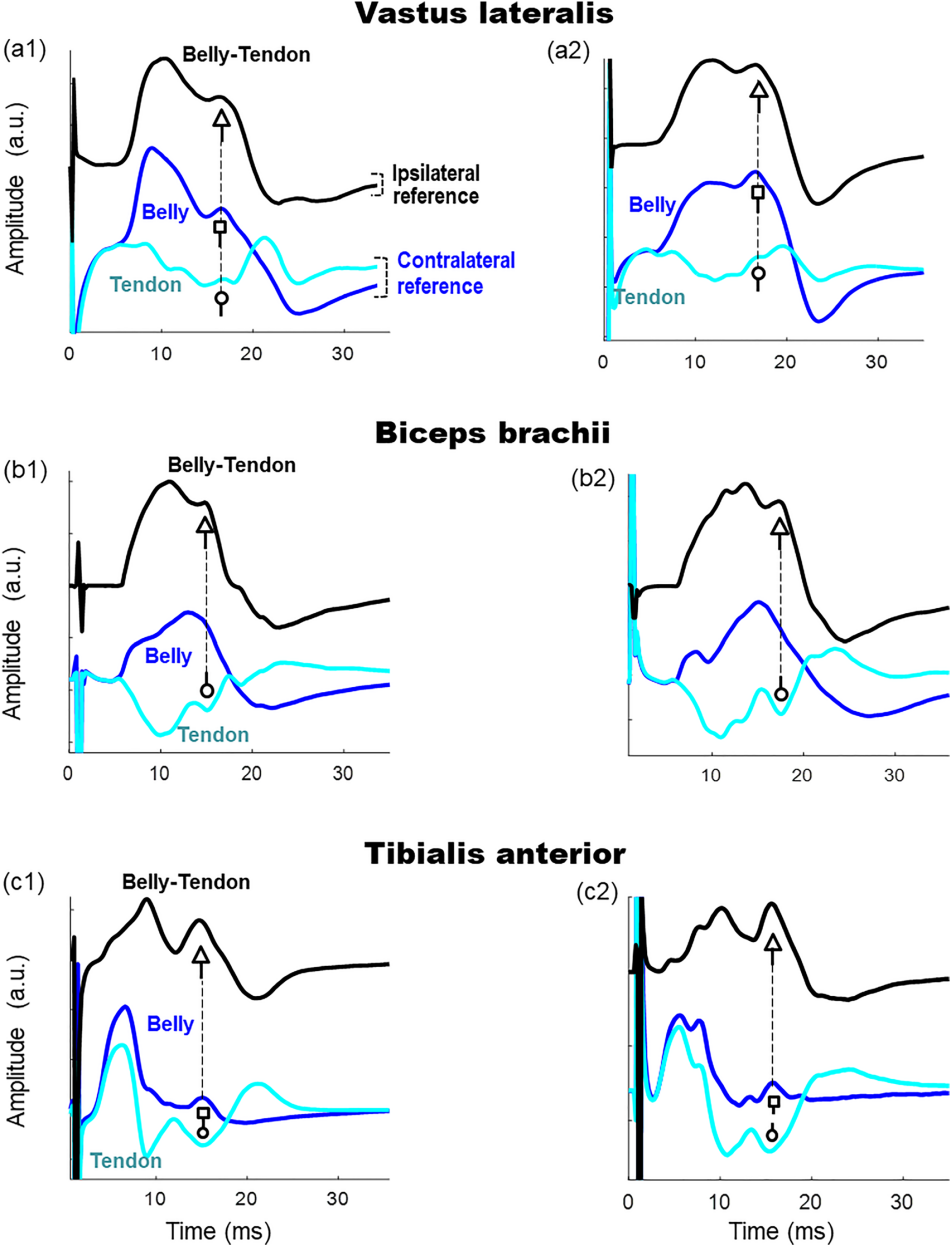
Representative examples of two belly–tendon M waves (upper black trace in each plot), together with their components at the belly and tendon sites (lower traces in each plot), recorded from two subjects in the vastus lateralis (a1 and a2), biceps brachii (b1 and b2), and tibialis anterior (c1 and c2). The white arrows indicate the shoulder (or second peak) in the belly–tendon M waves. The white squares indicate the presence of shoulders or peaks in the belly potential. The white circles indicate the presence of peaks in the tendon potential.

The next step was to determine whether the potential at the tendon site is solely responsible for the shoulder‐like feature in the belly–tendon M wave or the belly potential has any role in the shoulder generation. To address this point, we show, in each plot of Figure 5, the belly–tendon M wave at the top, aligned with the belly and tendon components at the bottom. It can be seen that the contribution of the tendon and belly potentials to the shoulder generation was different depending on the muscle.

In the vastus lateralis, the tendon potentials showed a peak or deflection (white circles in Figures 5a1 and a2) coinciding in latency with the shoulder of the belly–tendon M waves (white arrows): however, this tendon peak was of small amplitude and positive polarity. In this muscle, the belly potentials also exhibited a distinct shoulder (white squares in Figures 5a1 and a2) which was concurrent with the shoulder of the belly–tendon M waves (white arrows) (Table 2).

**TABLE 2:** Mean ± SD values of the latency of different M‐wave features (shoulder and peak) calculated for the tendon, belly and belly–tendon M waves recorded in the vastus lateralis, biceps brachii and tibialis anterior.

Muscle	BellyLatency_PEAK_ (ms)	Tendon Latency_PEAK_ (ms)	Belly–tendon Latency_SHOULDER_ (ms)
Vastus lateralis	16.9 ± 3.3	16.9 ± 3.0	16.7 ± 3.1
Biceps brachii	–	15.3 ± 3.7	15.2 ± 3.6
Tibialis anterior	14.3 ± 3.4	14.2 ± 3.6	14.5 ± 3.0

*n* = 38.

In the *biceps brachii*, the tendon potentials had an apparent negative peak (white circles in Figures 5b1 and b2), which was concurrent with the shoulder of the belly–tendon M waves (white arrows in Figures 5b1 and b2) (Table 2). In this muscle, the belly potentials did not have any shoulder in the declining first phase.

In the tibialis anterior, the tendon potential had a marked negative peak (white circles in Figures 5c1 and c2), and the belly potential had a positive peak (white squares in Figures 5c1 and c2), both peaks being concurrent with the shoulder of the belly–tendon M wave (white arrows in Figures 5c1 and c2) (Table 2).

## 4 DISCUSSION

The present study examined the contribution of the tendon electrode to the belly–tendon M waves of different muscles. The main findings were as follows: (1) The patellar tendon site showed only a low‐amplitude potential that contributed little to the belly–tendon M wave of the vastus lateralis, whereas, for the biceps brachii and tibialis anterior, the tendon potential showed a large amplitude, and thus had a great impact on the belly–tendon M wave, and (2) in the vastus lateralis, the tendon potential exhibited a small positive peak coinciding in latency with the shoulder of the belly–tendon M wave, while in contrast, in the biceps brachii and tibialis anterior, the tendon potential showed a clear negative peak which coincided in latency with the shoulder.

### 4.1 The contribution of the tendon potential to the belly–tendon M wave depends on the muscle and nerve stimulated

We found that the contribution of the tendon potential to the belly–tendon M wave varies depending on the muscle and nerve stimulated. Specifically, the patellar tendon potential recorded after femoral nerve stimulation was small in amplitude, and thus had little influence on the belly–tendon M wave of the vastus lateralis. It is for this reason that, in this muscle, the belly and belly–tendon M wave had rather similar amplitude and shape characteristics. In contrast, for the biceps brachii and tibialis anterior, the potentials at the tendon sites showed a large amplitude, and thus had a great impact on the final shape and magnitude of the corresponding belly–tendon M wave. Even so, the contribution of the tendon potential is different in these two muscles. In the biceps brachii, the tendon site had an ‘additive’ contribution to the belly–tendon M wave. Specifically, the initial negative phase of the tendon potential summated constructively with the first positive phase of the belly potential, and similarly, the final positive phase of the tendon potential acted to enlarge the negative phase of the belly potential (this can be appreciated in Figure 5b, 1 and b2). This ‘constructive’ contribution of the tendon potential explains why the belly–tendon M wave is greater in magnitude (+ ∼40%) than the belly M wave. However, in the tibialis anterior, the contribution of the tendon site was more complex. Because the belly and tendon potentials showed a rather similar initial positive phase (first 10 ms), this initial component was largely cancelled out when these potentials were subtracted to obtain the belly–tendon M wave (this cancellation can be appreciated in Figure 5c, 1 and c2). However, the final part of the belly potential (from 10 ms onwards) was rather flat and of low amplitude; thus, the tibialis anterior tendon site largely determined the shape and amplitude of the belly–tendon M wave for the final portion of this evoked signal.

### 4.2 The origin of the shoulder in the belly–tendon potential depends on the muscle and nerve stimulated

We found that the origin of the shoulder was different depending on the muscle. Namely, in the vastus lateralis, it was the belly potential, and not the tendon potential, which caused the shoulder in the belly–tendon M waves. Indeed, the tendon potential cannot be the cause of the shoulder for various reasons. First and most importantly, the peak in the tendon potential had positive polarity, and thus, when the tendon potential is subtracted from the belly potential, this positive peak would result in a negative peak (and not in a positive shoulder) in the belly–tendon M wave. Second, the positive peak in the tendon potential was too small in amplitude to generate the shoulder. Moreover, we found that the belly potential had itself a shoulder or second peak, and indeed, this peak was the actual generator of the shoulder in the belly–tendon M wave.

In the biceps brachii, by contrast, the tendon potential was the main cause of the shoulder. Indeed, in this muscle the tendon potential exhibited a distinct negative peak (white circles in Figure 5b, 1 and b2) that coincided in latency with the shoulder of the belly–tendon M wave, while the belly potential did now show any remarkable feature at this latency. Therefore, it is precisely this negative peak in the tendon potential which, after being subtracted from the belly potential, gave rise to the positive peak or shoulder in the belly–tendon M wave. Similarly, in the hypothenar muscles (ulnar nerve stimulation), previous studies have shown that the shoulder appearing in the belly–tendon M wave was caused by the negative phase of the tendon potential (Kincaid et al., 1993).

In the tibialis anterior, we found that both the negative peak of the tendon potential and the positive peak of the belly potential were concurrent with the shoulder of the belly–tendon M wave. Therefore, in this muscle both the tendon and belly potentials contributed to the shoulder generation in the belly–tendon M wave.

From the above, it could be concluded that the shoulder of the belly–tendon M wave is not due to the tendon potential in some muscles (vastus lateralis), whereas the tendon potential is the main generator of the shoulder in other muscles (biceps brachii and hypothenar muscles). Still, there are other muscles (tibialis anterior) in which both the tendon and belly potentials contribute to the shoulder. Thus, it must be stressed that the contribution of the tendon potential is not a necessary condition for the occurrence of the shoulder in the belly–tendon M waves, as suggested by Kincaid et al. (1993) and Nandedkar and Barkhaus (2007).

### 4.3 Why the tendon potential has different amplitude and shape depending on the nerve stimulated

Consideration will first be given to the factors that can affect the formation of the electrical potential at the tendon site. Subsequently, we will discuss how these factors come into play for each tendon site. Before entering the discussion, we must first remember some basic principles of bioelectricity: (1) EMG potentials essentially emerge from two bioelectric sources: quadrupoles and dipoles; (2) quadrupole sources only generate near‐field components, whereas dipole sources have both near‐field and far‐field components; and (3) near‐field potentials attenuate with the distance from the source, whereas far‐field potentials have the same amplitude irrespective of the distance from the source (Stegeman et al., 1997).

The first factor to consider is the distance between the tendon electrode and the muscle(s) activated by peripheral nerve stimulation. It is normally argued that, because the tendon electrode is placed beyond the end of a muscle (typically over a tendon or bone), this electrode can only capture far‐field electrical activity (Nandedkar & Barkhaus, 2007). However, if the tendon site is close enough to the activated muscle, then this electrode may also pick up near‐field potentials. The second factor to consider is the spatial configuration of the muscles (i.e., how are they oriented in respect to the main distal–proximal axis) and the fibre arrangement of each muscle (fusiform, pennate, etc.). Indeed, these geometrical aspects could determine whether electrical fields from the different muscles summate additively or destructively at the tendon site (Van Dijk et al., 1999). The third aspect is the muscle mass innervated by the stimulated nerve. Indeed, the greater the innervated muscle mass, the higher the number of dipoles created at the fibre ends by the extinction of action potentials, and thus the larger the far‐field component at the tendon site (Nandedkar & Barkhaus, 2007). A final important factor refers to the geometric and conductivity characteristics of the volume conductor (Dumitru & King, 1991). This is a key aspect since virtual dipoles (and consequently far‐field potentials) can be created when the moving source encounters a change/transition in the geometry and/or conductivity of volume conductor (Stegeman et al., 1997).

The low amplitude of the potential recorded at the patellar tendon after stimulation of the femoral nerve (compared to the belly potential) could be due to the fact that the muscles innervated by this nerve (vastus lateralis, vastus medialis, vastus intermedius and rectus femoris) are oriented so that the electrical activity arising from these muscles may summate destructively at the patellar tendon. In favour of this theory of the partial cancellation of the electrical activity is the high number of phases present in the patellar tendon potential (tri‐ or quatriphasic waveform, see Figure 3a3). In contrast, the potential recorded at the antecubital fossa after brachial plexus stimulation had an amplitude comparable to the belly potential (at the biceps brachii). Various factors may explain this finding. First, the electrical activity arising from the muscles innervated by the brachial plexus may summate constructively at the antecubital fossa. Second, the antecubital fossa is in close proximity to many muscles (biceps brachii, brachialis, brachioradialis), and thus it is likely that the tendon electrode picks up near‐field electrical activity from the dipoles created by the extinction of action potentials at the fibre ends.

The potential recorded at the tibialis anterior tendon site after peroneal nerve stimulation showed an unusual shape, and thus deserves special attention. Indeed, this tendon potential shows a triphasic waveform (positive–negative–positive, see Figure 3c3), and not the well‐known biphasic waveform (initial long negative phase followed by a positive phase) that would be expected according to the classic volume‐conductor theory (Gydikov & Kosarov, 1972; Rodriguez et al., 2012). Clearly, the first positive phase of this tendon potential, appearing immediately after the stimulus artifact, is the odd one. Interestingly, this short‐latency positive phase at the tendon site coincided in latency with the first positive phase of the belly potential, thus indicating that this positive component has a standing (non‐propagating) character. In other words, this initial positive phase would be generated by a ‘stationary’ dipole source.

### 4.4 The shoulder (peak) in the belly potential of the vastus lateralis and tibialis anterior

One surprising finding of the present study is that the potential recorded at the belly of the vastus lateralis and tibialis anterior exhibited a distinct shoulder (or peak) in the first phase. Whereas it is difficult to determine the exact electrical origin of this shoulder, one key common observation was made in both muscles: the shoulder (or peak) in the belly potential occurred at the same latency as a peak in the tendon potential. This observation unequivocally indicates that this shoulder feature had a standing (non‐propagating) character, and therefore was necessarily generated by a ‘stationary’ electrical source, that is, a dipole (Stegeman et al., 1997). Now, the question arises as to how this dipole was created. It would be tempting to propose that such a dipole was generated upon the extinction of action potentials at the fibre–tendon junctions of the vastus lateralis and tibialis anterior (Gydikov & Kosarov, 1972). However, bioelectrical theory predicts that such end‐of‐fibre potentials present a negative polarity at the belly of the muscle (Dumitru & King, 1991), which is opposite to the positive shoulder (peak) observed in the belly potential. Another possibility is that the dipole was created by the extinction of action potentials at the fibre ends of other ‘neighbouring’ muscles innervated by the femoral nerve and peroneal nerve (different from the vastus lateralis and tibialis anterior). Finally, the dipole could be created as the moving source reaches a transition in the volume conductor geometry/conductivity/morphology (Kimura et al., 1984).

### 4.5 Practical implications and recommendations

The present work issues a warning on the stability of the belly–tendon M waves (recorded in some muscles) throughout an experimental session. Because the potentials at the tendon sites of the biceps brachii and tibialis anterior make a great contribution to the belly–tendon M wave, it is likely that the changes in the amplitude and shape observed in the belly–tendon M waves of these muscles are partly due to alterations in the activity at the tendon electrode, and not only to changes at the belly electrode (Calder et al., 2005). Thus, in these muscles it would be advisable to record M waves in bipolar configuration to improve stability: indeed, this is the configuration already adopted by some authors (Booghs et al., 2012; Levenez et al., 2008; Riley et al., 2008). In contrast, in the vastus lateralis, the patellar tendon contribution is minimal compared to the belly electrode signal, and thus changes in tendon potential would hardly impact the stability of the belly–tendon M wave. Thus, the belly–tendon configuration can continue to be safely used in the vastus lateralis.

The contribution of the tendon electrode may also be of importance for the determination of the stimulation intensity that ensures full motor unit recruitment. In this sense, it was previously found that, as stimulation intensity is gradually increased, the belly–tendon M wave reaches the plateau at a significantly higher stimulus intensity compared to the bipolar M wave (Rodriguez‐Falces & Place, 2016). In the light of the present findings, such a difference in the maximal stimulation intensity could be due to the fact that, in the belly–tendon configuration, the tendon potential continues increasing in amplitude due to far‐field activity, even when all motor units are already recruited. This ‘misleading’ estimation of the maximal stimulation intensity with the belly–tendon M waves would have a greater impact on the biceps brachii and tibialis anterior muscles, where the contribution of the tendon electrode is prominent.

The present experiments were conducted only in male participants. Thus, this single‐sex sample should be acknowledged as a limitation since the results obtained from males may not be generalizable to females.

In view of the present findings the assumption that ‘the first phase of the belly–tendon M wave results from the propagation of the action potentials along muscle fibres, whereas the second reflects the extinction of these action potentials at the tendon’ proposed by Rodriguez‐Falces et al. (2022) should be revisited. Indeed, here we showed that the belly potential of the vastus lateralis (and tibialis anterior) exhibited a distinct shoulder (or peak) in the first phase, which had a non‐propagating character. Whether this shoulder is caused by end‐of‐fibre signals emerging from the vastus lateralis or from another ‘neighbouring’ muscle is still to be elucidated. Nevertheless, the above assumption should be refined by saying that, in belly–tendon M waves only the portion of the first phase that is between the onset and the shoulder‐like feature can be regarded as having a propagating character.

Another unexpected finding was that the tendon potential of the tibialis anterior started with a large positive phase of short latency, and not with an initial negative phase predicted by bioelectricity fundamentals (Dumitru & King, 1991). Not only that, this early positive phase of the tendon potential coincided in latency with the positive phase of the belly potential. We, therefore, postulate that this initial positive phase has non‐propagating character and corresponds to the so‐called short‐latency component previously reported by Kincaid et al. (1993). In any case, this short‐latency positive phase is largely cancelled when the belly and tendon potentials are subtracted to obtain the belly–tendon M wave.

## 5 CONCLUSIONS

Two main conclusions can be drawn from this study: (1) because the patellar tendon potential is small in amplitude, it hardly influences the amplitude and shape of the belly–tendon M wave of the vastus lateralis: however, for the biceps brachii and tibialis anterior muscles, the potentials at the tendon sites show a large amplitude, and thus have a great impact on the corresponding belly–tendon M waves, and (2) the shoulder observed in the belly–tendon M wave of the vastus lateralis is caused by the belly potential, the shoulder in the biceps brachii M wave is generated by the tendon potential, and the shoulder in the tibialis anterior M wave is caused by both the tendon and belly potentials.

Two unexpected findings must be noted: (1) in the vastus lateralis, the belly potential has itself a shoulder or second peak, and (2) the potential recorded at the tibialis anterior tendon site has a prominent short‐latency positive phase.

Two main messages emerge from this study: (1) the contribution of the tendon electrode to the belly–tendon M wave depends greatly on the nerve/muscle stimulated, and (2) the assumption that the ‘belly–tendon shoulder is present only in those muscles where the tendon potential makes a significant contribution to the belly–tendon M wave’ is incorrect.

## AUTHOR CONTRIBUTIONS

Javier Rodriguez‐Falces, Saioa Etxaleku, Gabriel S. Trajano and Igor Setuain contributed to the conception and design of the study. All authors were involved in the acquisition, analysis or interpretation of data for the work. All authors were involved in drafting the work or revising it critically for important intellectual content. Additionally, all authors approved the final version of the manuscript and agree to be accountable for all aspects of the work in ensuring that questions related to the accuracy or integrity of any part of the work are appropriately investigated and resolved. All persons designated as authors qualify for authorship, and all those who qualify for authorship are listed.

## CONFLICT OF INTEREST

The authors declare no conflicts of interest.

## Data Availability

The data that support the findings of this study are available from the authors upon reasonable request.
